# Increased expression of fibroblast growth factor 23 is the signature of a deteriorated Ca/P balance in ageing laying hens

**DOI:** 10.1038/s41598-020-78106-7

**Published:** 2020-12-03

**Authors:** A. Gloux, N. Le Roy, N. Même, M. L. Piketty, D. Prié, G. Benzoni, J. Gautron, Y. Nys, A. Narcy, M. J. Duclos

**Affiliations:** 1 INRAE, Université de Tours, UMR BOA, 37380 Nouzilly, France; 2 Service des Explorations Fonctionnelles, G.H. Necker Enfants Malades, Université Paris Descartes Faculté de Médecine, INSERM U11513, 75743 Paris Cedex 15, France; 3 ADM Animal Nutrition, Talhouët, 56250 Saint-Nolff, France

**Keywords:** Transcription, Calcium and vitamin D

## Abstract

The present study concerned the effect of ageing in laying hens, from 23 to 90 weeks of age, on the regulation of Ca metabolism related to the requirement for eggshell mineralization. Samples were collected from parathyroid gland (PG), liver, jejunum, medullary bone (MB) and kidney for a quantitative study of candidate gene expression. Although parathyroid hormone (*PTH*) gene expression in the PG did not vary with age, a stronger challenge to Ca homeostasis was suggested in aged hens. Indeed gene expression of Ca transporters , Vitamin D Receptor (*VDR)* in the jejunum, and that of transient receptor potential channel subfamily V member 5 (*TRPV5*) in the kidney decreased. This could exacerbate bone resorption and impair bone accretion, as attested by a higher expression of the Carbonic Anhydrase 2 (*CA2*) gene and a lower expression of collagen type I alpha 1 chain (*COL1A1*) in the MB. The increased expression of Fibroblast Growth Factor 23 (*FGF23*) in the MB likely contributed to the decreased plasma levels of 1.25(OH)_2_D_3_ and the altered expression of target genes under its regulation. Our data highlights the molecular mechanisms underlying the osteoporotic syndrome previously documented in aged laying hens, thus providing new perspectives for future interventions.

## Introduction

The egg industry is facing a huge demand world-wide and plans to increase the number of eggs produced per hen, by extending the egg production period from 85 to 100 weeks of age^1^. However, laying hens are prone to osteoporosis with ageing, a major problem leading to both welfare issues and financial losses^2,3^. Indeed, for each egg laid the hen exports 2 g of calcium (Ca) originating from its diet to allow for the mineralization of the eggshell. As the hen feeds during the day and mineralizes its egg during the night, this sets the need for an intermediate store of Ca in its skeleton^4^. This relies on the medullary bone, which develops at sexual maturity and constitutes a rapidly mobilizable source of Ca^3^, with a daily cycle of bone turnover^5,6^. Following oviposition, the mineralizing activity is minimal and bone accretion occurs. The eggshell mineralization phase, which takes from 9 to 10 h, induces a high demand for Ca, which is linear until 2 h before the next oviposition. The mineralizing activity increases linearly during this phase, being intermediate at 9–10 h and maximal at 18–19 h post ovulation (PO), stimulating osteolysis of the MB^4^. With repeated solicitations, the proportion of cortical bone, which ensures bone strength, decreases with age as it is replaced by medullary bone^3^. The later exhibits a different biochemical composition of its organic matrix^7^. These modifications make the bone more fragile and prone to fractures, which were originally described in layer flocks from the end of the peak of lay and qualified as osteoporosis^8,9^. A recent study indicates that fractures are detectable by radiography of the keel bone from 35 weeks of age, in caged or floor-housed hens^10^. Additionally, the reduced eggshell quality at the end of the egg production period^11,12^, affecting the number of saleable eggs is currently the main reason to terminate a layer flock^13^. The low eggshell solidity can be partly attributed to the increase in egg weight, without an increase of calcium carbonate (CaCO_3_) deposition^12,14,15^, and to disorders in vitamin D metabolism^16^. All this sets the need for a better characterization of the mechanisms underlying Ca metabolism to reach the objective of extending the duration of egg production, reducing osteoporosis while maintaining the eggshell quality.

In old compared to young hens, a decrease in circulating 1.25(OH)_2_D_3_ plasma levels, a reduced activity of the 1-α hydroxylase enzyme^11,16^ and a reduced intestinal Ca absorption have been observed^12,17^. Although alterations in the bone structure have been reported, the molecular mechanisms involved have not been studied so far. We recently identified the coordinated response of candidate genes regulating Ca homeostasis across tissues in young laying hens compared at different stages of the ovulatory cycle with minimal, intermediate or high mineralizing activity^18^. The high demand of Ca by the uterus for eggshell formation activates the Parathyroid Hormone (*PTH*) gene and the genes under its control involved in medullary bone resorption. The expression of the phosphaturic hormone Fibroblast Growth Factor 23 (*FGF23*) in the MB starts at the initiation of eggshell formation and rises to a maximum during the active phase of eggshell mineralization, then turns of following oviposition. Its product, FGF23 likely triggers the urinary excretion of P through reduced expression of the renal P cotransporters and the return to normophosphatemia after oviposition^18^. It could also induce the decrease of 1.25(OH)_2_D_3_ plasma levels by the up regulation of the 24 hydroxylase gene in the kidney^18^. As active immunization of hens against FGF23 increases P retention^19^, these observations highlight this factor as an important regulator of the Ca/P balance in laying hens.

Therefore, the objective of the present work was to study the effect of laying hens’ ageing on those regulations of Ca metabolism related to the requirement for eggshell mineralization. For this, we compared hens at maximum of lay and at end of lay (23 versus 90 weeks of age). We qualify these hens as young and old, although 90 weeks remain an early stage when *Gallus gallus* has a life expectancy of 10–12 years.

The first hypothesis was that an imbalance in the regulation of Ca metabolism could occur in old hens leading to reduced Ca retention and enhanced bone resorption.

The second hypothesis was that *FGF23* could be overexpressed in old hens and act as a signal decreasing plasma 1.25(OH)_2_D_3_ and the expression of genes under its control.

## Results

### Effect of ageing on eggshell quality parameters

**Figure 1:**
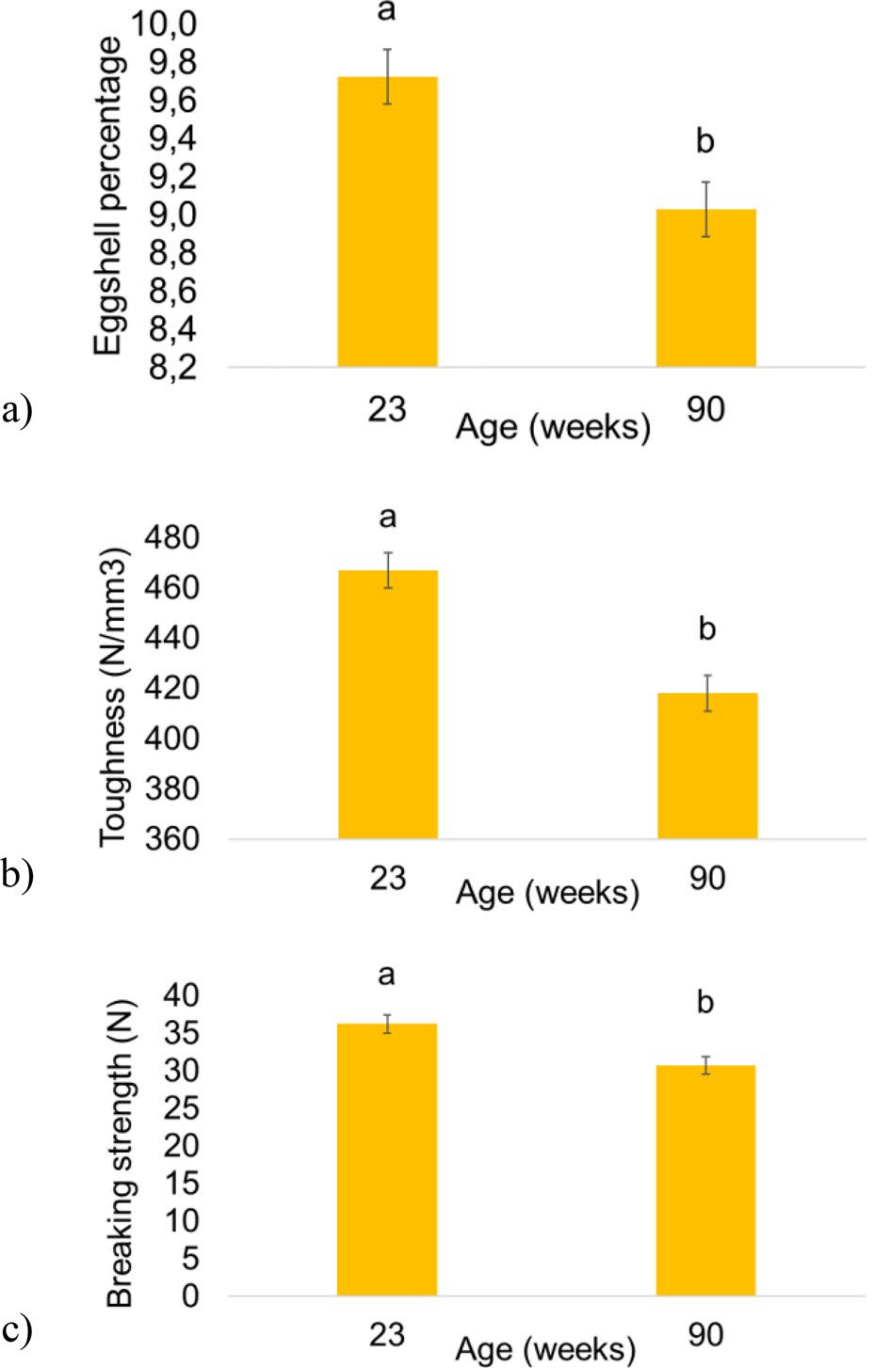
Effect of the age of hens on eggshell quality parameters. (**a**–**c**) Data are expressed as mean ± SEM with n = 87–89. Different letters indicate significant differences (*p* < 0.05).

All three parameters of eggshell quality (Fig. 1) significantly decreased with age (*p* < 0.05). The eggshell percentage was lower in old (90 weeks of age) than in young hens (23 weeks of age) (9.03 ± 0.14% and 9.73 ± 0.14%, respectively, Fig. 1a). A lower toughness was observed at 90 than at 23 weeks of age (418.1 ± 6.02 and 467.0 ± 7.12 N/mm^3^, respectively, Fig. 1b). The breaking strength was also lower at 90 than at 23 weeks of age (30.7 ± 1.21 and 36.3 ± 1.23 N, respectively, Fig. 1c). These observations, which are in agreement with earlier studies, validate our experimental set-up. We then assessed the classical plasma parameters of Ca/P metabolism and conducted an expressional study concerning 40 selected genes across 5 tissues involved in its regulation.

### Plasma levels of *i*P, vitamin D metabolites, and PTH expression in the parathyroid gland

Total plasma Ca (226–252 mg/l) varied neither with the PO stage, nor with the age (*p* = 0.7 for stage PO, *p* = 0.45 for age). The effect of the PO stage and the age on plasma levels of 25(OH)D_3_, 1.25(OH)_2_D_3_ and *i*P are shown in Table 1. No interaction was observed between the PO stage and the age for any parameter. The plasma levels of 25(OH)D_3_ neither varied with the PO stage nor with the age of the hens. By contrast the plasma levels of 1.25(OH)_2_D_3_ increased between 0–1 h and 18–19 h PO, when the mineralizing activity is known to increase. They were higher at 23 than at 90 weeks of age (*p* < 0.01 and *p* < 0.001, respectively). Plasma *i*P was higher during the active phase of eggshell mineralization (18–19 h PO) than during rest or initialization of mineralization at 0–1 and 9–10 h PO respectively (*p* < 0.001), but was not affected by the age. In the parathyroid gland, the variations of the parathyroid hormone (*PTH*) gene mRNA expression paralleled that of plasma iP (*p* < 0.01). No variation with the PO stage or the age was detected for the mRNA expression of the Ca sensing receptor (*CaSR*).

**Table 1:** Effect of the post-ovulation stage and the age on plasma levels of vitamin D metabolites and *i*P and the mRNA expression of genes in the parathyroid gland (PG).

	Plasma levels				mRNA expression (AU)	
	25(OH)D_3_ (ng/ml)	1.25(OH)_2_D_3_ (pg/ml)	Ca (mg/l)	*i*P (mg/l)	PTH	CaSR
	P values					
**PO stage**						
0–1	42.9 ± 4.8	275^b^ ± 22.6	229 ± 10	108.8^b^ ± 4.87	1.43^b^ ± 0.33	1.32 ± 0.18
9–10	46.1 ± 5.1	343.3^a,b^ ± 23.9	240 ± 11	123.6^b^ ± 5.28	1.67^b^ ± 0.31	1.81 ± 0.17
18–19	49.4 ± 5.0	375.3^a^ ± 23.6	234 ± 11	147.1^a^ ± 5.10	2.71^a^ ± 0.30	1.45 ± 0.16
**Age**						
23	42.3 ± 3.8	388.1^a^ ± 18.0	229 ± 8	123.8 ± 3.88	2.13 ± 0.23	1.46 ± 0.13
90	50.0 ± 4.3	274.3^b^ ± 20.3	239 ± 9	129.3 ± 4.41	1.75 ± 0.28	1.59 ± 0.15
PO stage	0.356	0.006	0.728	< .0001	0.008	0.584
Age	0.200	0.000	0.457	0.481	0.314	0.507

Values are presented as LSMeans ± SEM with n = 11–12 per post-ovulation stage and n = 17–19 per age. For PTH and CaSR, n = 8–10 per post-ovulation stage and n = 11–16 per age. PO stage = post-ovulation stage (hour).

^a,b^Means with different superscripts for each effect tested in each model are significantly different (*P* < 0.05).

### Candidate genes in the bone

**Figure 2:**
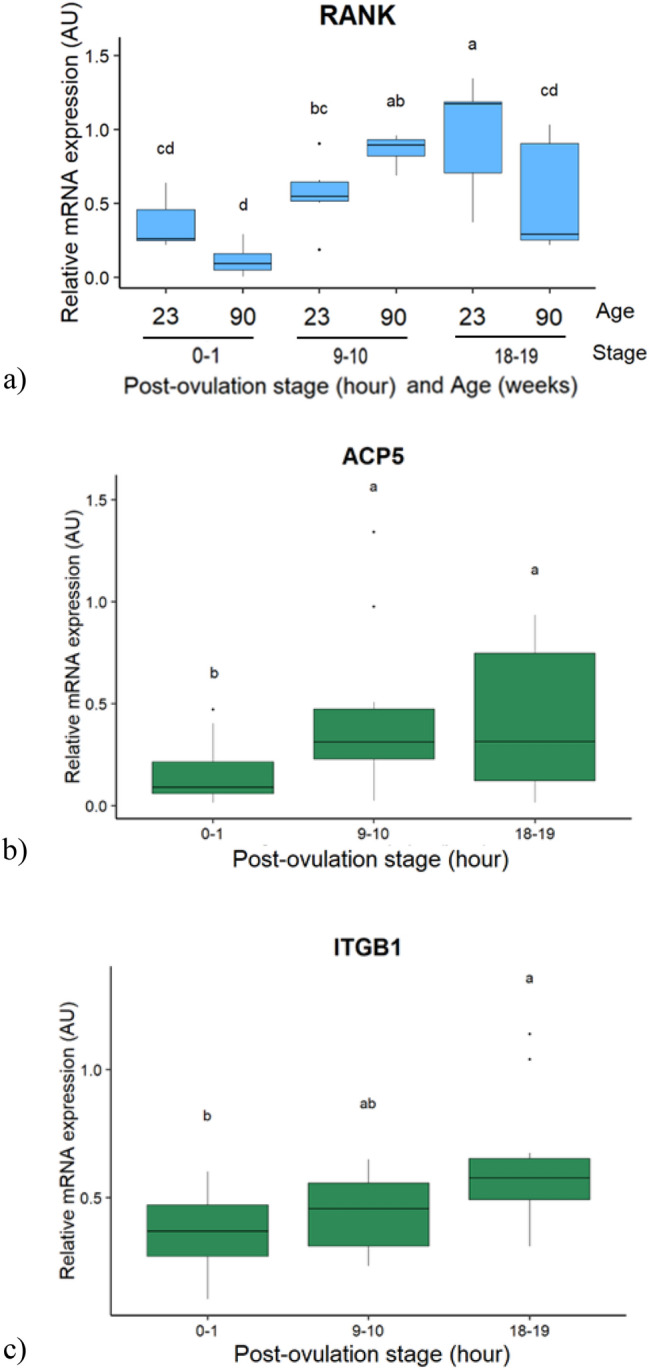
Boxplot of the interaction effect between the post-ovulation stage and the age of hens, or the post-ovulation stage effect on the relative mRNA expression of candidate genes in the medullary bone. (**a**) Receptor activator of nuclear factor-kappaB (RANK). (**b**) Acid phosphatase 5, tartrate resistant (ACP5). (**c**) Integrin subunit beta 1 (ITGB1). Values are expressed as Arbitrary Units (AU) with n = 4–7 per group in (**a**) and n = 15–20 per post-ovulation stage in (**b**) and (**c**). Different letters indicate significant differences (*p* < 0.05).

Eleven genes were selected and studied because of their functions in medullary bone remodeling (see supplementary table). The mRNA expression levels of *RANKL*, *CTSK* and *MMP2* did not vary with the PO stage or the age (data not shown). The genes that were affected by the interaction between the age and the PO stage are shown in Fig. 2 (*p* < 0.05). The mRNA expression of *RANK*, the receptor for *RANKL*, which enhances osteoclastogenesis, was altered by the interaction between the PO stage and the age, so that it was higher in young than in old hens, only at 18–19 h PO (Fig. 2a). In addition, while in young hens, its expression increased between 9–10 h and 18–19 h PO, in old hens it increased between 0–1 and 9–10 h PO.

The Acid Phosphatase 5 tartrate resistant (*ACP5*) is involved in bone resorption, and the Integrin Subunit Beta 1 (*ITGB1*) is an actor of bone remodeling. In both groups of hens, the former was upregulated at 9–10 h and 18–19 h compared to 0–1 h PO (Fig. 2b), and the latter at 18–19 h PO compared to 0–1 h PO (Fig. 2c).

**Figure 3:**
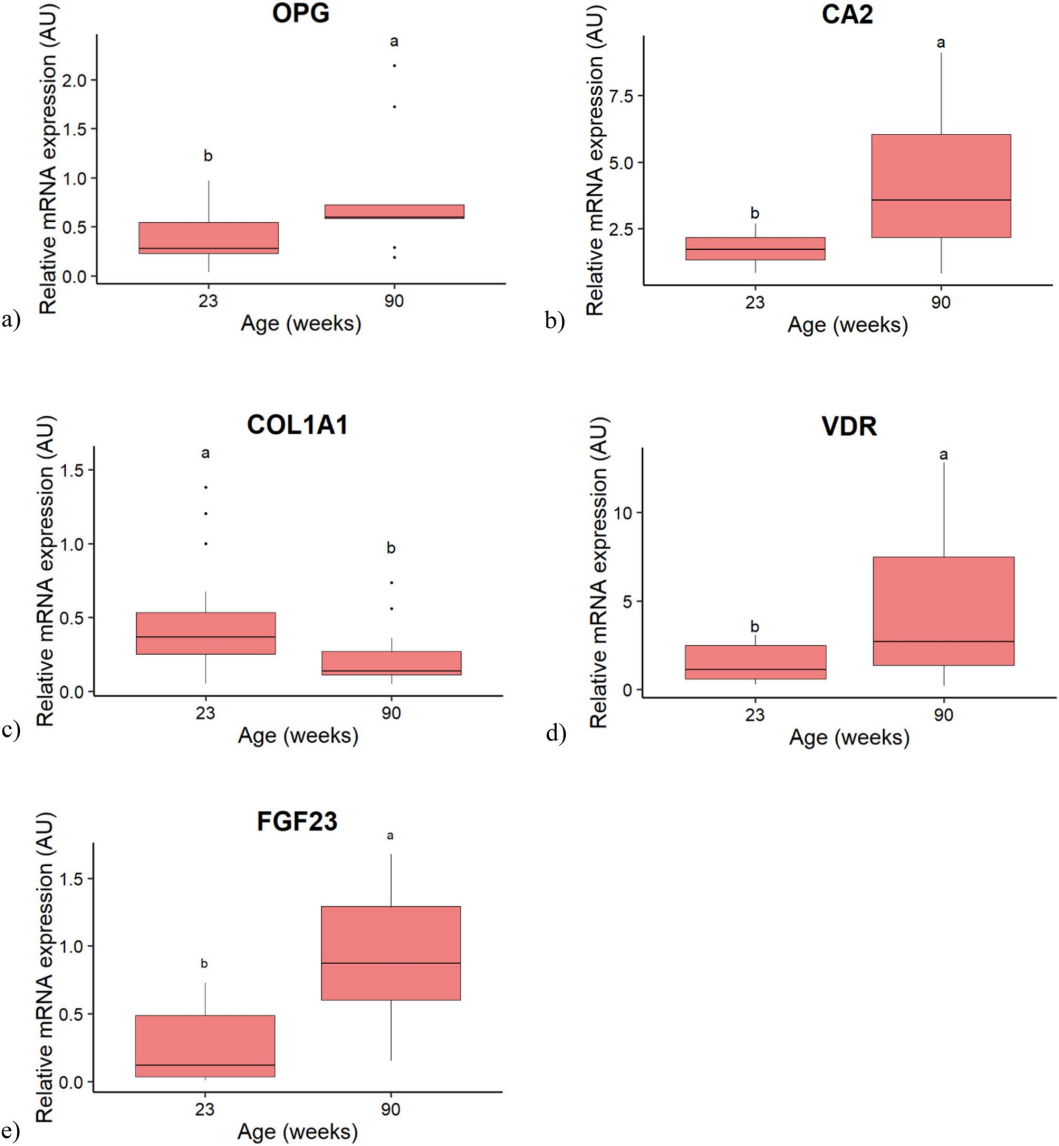
Boxplot of the age effect on the relative mRNA expression of candidate genes in the medullary bone of hens. (**a**) Osteoprotegerin (OPG). (**b**) Carbonic anhydrase 2 (CA2). (**c**) Collagen type I alpha 1 chain. (**d**) Vitamin D receptor (VDR). (**e**) Fibroblast growth factor 23 (FGF23). Values are expressed as Arbitrary Units (AU) with n = 11–12 per age for (**b**), (**c**), (**d**) and n = 9–14 per group for (**a**) and (**e**). Different letters indicate significant differences (*p* < 0.05).

The genes affected by the age are presented in the Fig. 3 (*p* < 0.05). The expression of the phosphaturic hormone *FGF23* and of the inhibitor of osteoclastogenesis, osteoprotegerin (*OPG*) were below the detection levels for the samples at 0–1 h PO, so that only the stages 9–10 and 18–19 h PO were considered in the statistical analysis. The mRNA expressions of the Carbonic Anhydrase 2 (*CA2*), an enzyme involved in bone resorption, of the Vitamin D receptor (*VDR*) and Collagen type 1 alpha 1 chain (*COL1A1*), involved in bone accretion, were measurable in all samples. The mRNA expressions of *OPG*, *CA2*, *VDR* and *FGF23* increased with age (Fig. 3a, b, d, e, respectively), while that of *COL1A1* decreased (Fig. 3c).

### Expression of genes involved in renal reabsorption or jejunal absorption of Ca and P

**Figure 4:**
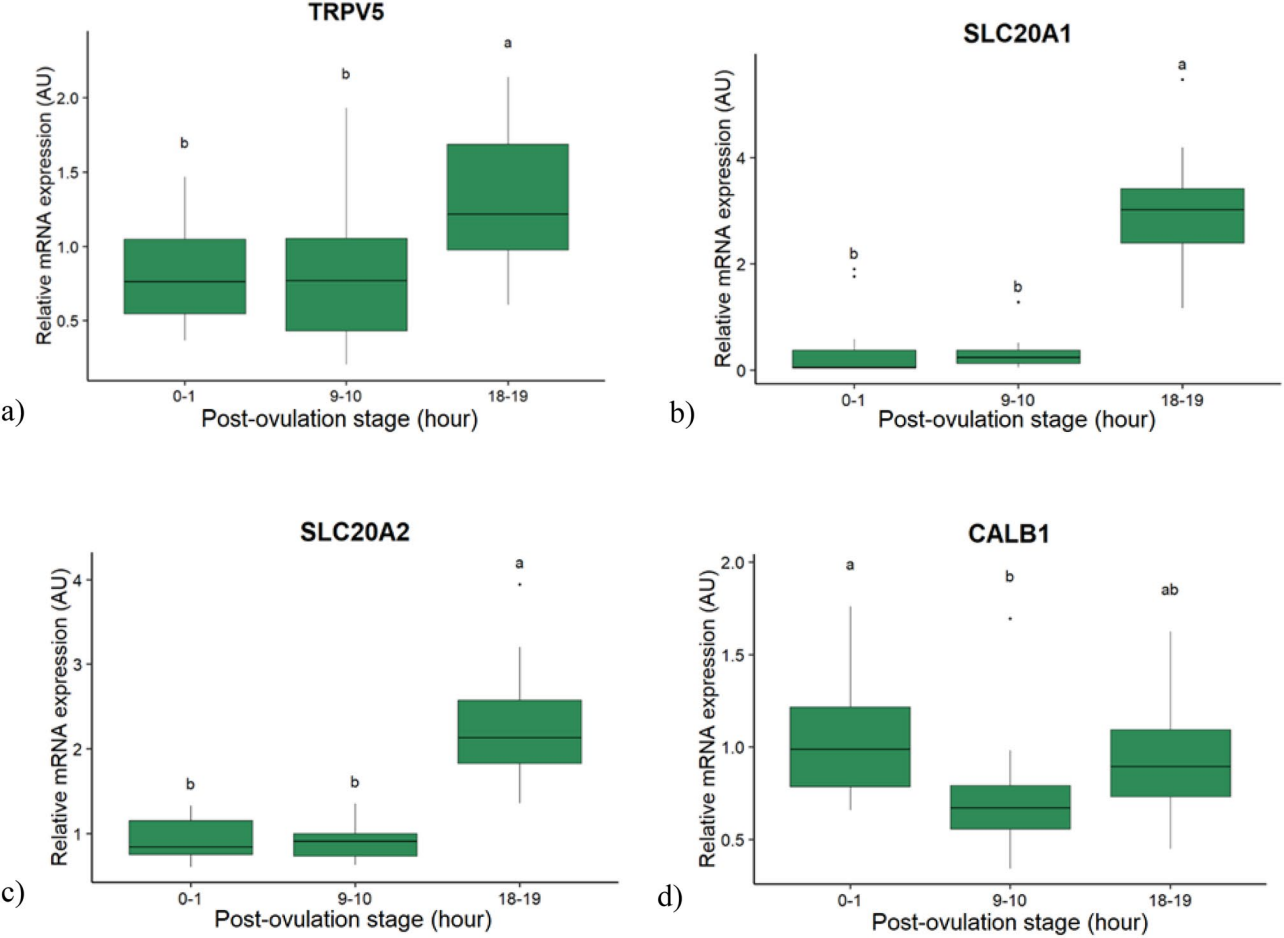
Boxplot of the post-ovulation stage effect on the relative mRNA expression of candidate genes in the kidney of hens. (**a**) Transient receptor potential cation channel subfamily V member 5 (TRPV5). (**b**) Solute carrier family 20 member 1 (SLC20A1). (**c**) Solute carrier family 20 member 2 (SLC20A2). (**d**) Calbindin 1 (CALB1). Values are expressed as Arbitrary Units (AU) with n = 15–20 per post-ovulation stage. Different letters indicate significant differences (*p* < 0.05).

**Figure 5:**
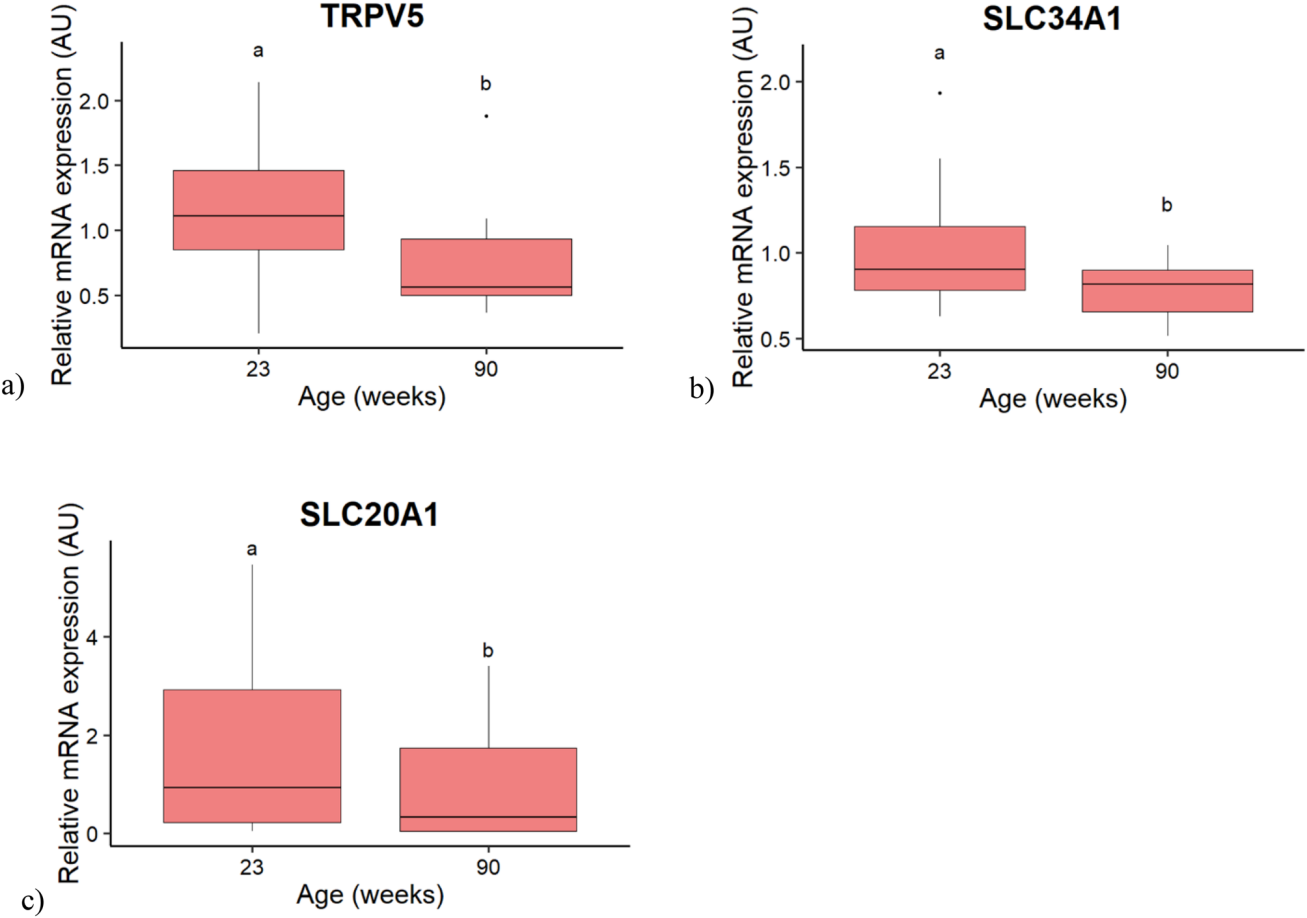
Boxplot of the age effect on the relative mRNA expression of candidate genes in the kidney of hens. (**a**) Transient receptor potential cation channel subfamily V member 5 (TRPV5). (**b**) Solute carrier family 34 member 1 (SLC34A1). (**c**) Solute carrier family 20 member 1 (SLC20A1). Values are expressed as Arbitrary Units (AU) with n = 11–12 per age. Different letters indicate significant differences (*p* < 0.05).

In the kidney, 2 candidate genes involved in Ca or P reabsorption were affected only by the PO stage (*CALB1* and *SLC20A2*), 1 only by the age (*SLC34A1*) and 2 by both factors (*TRPV5* and *SLC20A1*) without interaction (*p* < 0.05). Four genes (*ATP2B1, KL*, *FGFR2* and *FGFR3*) varied neither with the PO stage, nor with the age of the hens. The 4 candidate genes influenced by the PO stage are presented in the Fig. 4. The mRNA expression of the Ca channel *TRPV5* and the Sodium-phosphate symporters *SLC20A1* and *SLC20A2* were higher at 18–19 h PO compared to 0–1 and 9–10 h PO (Fig. 4a–c). The expression of the Ca-binding protein *CALB1* was higher at 0–1 than at 9–10 h PO, and intermediate at 18–19 h PO (Fig. 4d). The expression levels of the 3 genes affected by the age, *TRPV5*, *SLC34A1* and *SLC20A1*, decreased between 23 and 90 weeks of age (Fig. 5).

In the jejunum, out of the sixteen genes involved in Ca^2+^ and P absorption, 3 (*ATP2B2*, *CLDN12*, *OCLN*) were affected by the interaction between the age and the PO stage (Table 2). Nine genes (*VDR*, *TRPM7*, *TRPC1*, *TRPV2*, *ATP2B4*, *CLDN2*, *TJP1*, *TJP2, SLC34A2*) were influenced by the stage or the age independently (Table 3) and four genes (*CALB1*, *ATP2B1*, *TJP2*, *SLC20A1*) neither varied with the stage nor with the age.

**Table 2:** Effect of interaction between the post-ovulation stage and the age on the relative mRNA expression of candidate genes for the transcellular and paracellular pathways involved in Ca absorption in the jejunum.

	Genes	ATP2B2*	CLDN12^†^	OCLN^†^
Age	PO stage			
23	0–1	0.16 (± 0.09)^b^	0.72 (± 0.07)^b^	0.90 (± 0.06)^a^
	9–10	0.69 (± 0.09)^a^	1.26 (± 0.07)^a^	0.62 (± 0.06)^b^
	18–19	0.43 (± 0.09)^ab^	0.76 (± 0.06)^b^	0.71 (± 0.06)^ab^
90	0–1	0.18 (± 0.09)^b^	0.56 (± 0.07)^b^	0.61 (± 0.06)^b^
	9–10	0.30 (± 0.13)^ab^	0.61 (± 0.10)^b^	0.58 (± 0.09)^b^
	18–19	0.17 (± 0.11)^b^	0.52 (± 0.08)^b^	0.76 (± 0.07)^ab^
		Source of variation		
Age		0.864	0.092	0.002
PO stage		0.040	0.731	0.026
Age × PO stage		0.002	< .0001	0.003

*Genes of the transcellular pathway; ^†^genes of the paracellular pathway. PO stage = post-ovulation stage (hour). Relative expression values expressed as Arbitrary Units are presented as LSMeans (± SEM) with n = 10–12 per PO stage; n = 15–19 per age and n = 4–6 per PO stage and age.

^a,b^Means with different superscripts for each effect tested in each model are significantly different (P < 0.05). ATPase plasma membrane Ca^2+^ transporting 2 (ATP2B2); Claudin 12 (CLDN12); Occludin (OCLN).

**Table 3:** Effect of the post-ovulation stage and the age of hens on the relative mRNA expression of candidate genes for the transcellular, paracellular pathways involved in Ca absorption in the jejunum.

	PO stage			Age		Source of variation	
	0–1	9–10	18–19	23	90	PO stage	Age
VDR	1.38 (± 0.11)	1.22 (± 0.13)	1.60 (± 0.11)	2.34 (± 0.08)^a^	0.45 (± 0.10)^b^	0.140	< .0001
TRPM7*	0.80 (± 0.07)	0.77 (± 0.08)	0.62 (± 0.07)	0.82 (± 0.05)^a^	0.64 (± 0.06)^b^	0.077	0.038
TRPC1	0.74 (± 0.06)^a^	0.65 (± 0.08)^ab^	0.42 (± 0.06)^b^	0.48 (± 0.05)^b^	0.73 (± 0.06)^a^	0.002	0.004
TRPV2*	1.45 (± 0.28)	0.97 (± 0.33)	1.41 (± 0.29)	2.16 (± 0.22)^a^	0.39 (± 0.27)^b^	0.933	< .0001
ATP2B4*	0.56 (± 0.04)	0.59 (± 0.05)	0.64 (± 0.04)	0.46 (± 0.04)^b^	0.74 (± 0.04)^a^	0.218	< .0001
CLDN2^†^	0.79 (± 0.10)	0.63 (± 0.12)	0.97 (± 0.10)	1.12 (± 0.08)^a^	0.47 (± 0.10)^b^	0.216	< .0001
TJP1^†^	0.76 (± 0.07)	0.76 (± 0.09)	0.61 (± 0.07)	0.57 (± 0.06)^b^	0.86 (± 0.07)^a^	0.169	0.003
TJP3^†^	0.84 (± 0.12)	0.98 (± 0.14)	1.10 (± 0.12)	1.14 (± 0.09)^a^	0.81 (± 0.11)^b^	0.121	0.030
SLC34A2^‡^	2.07 (± 0.21)	2.19 (± 0.24)	2.07 (± 0.21)	3.71 (± 0.17)^a^	0.52 (± 0.20)^b^	0.978	< .0001

*Genes of the transcellular pathway; ^†^genes of the paracellular pathway; ^‡^P transporters. PO stage = post-ovulation stage (hour). Relative expression values expressed as Arbitrary Units are presented as LSMeans (± SEM) with n = 10–12 per PO stage; n = 15–19 per age.

^a,b^Means with different superscripts for each effect tested in each model are significantly different (*p* < 0.05). Vitamin D Receptor (VDR); Transient receptor potential cation channel subfamily M member 7 (TRPM7); Transient receptor potential cation channel subfamily C member 1 (TRPC1); Transient receptor potential cation channel subfamily V member 2 (TRPV2); calbindin 1 (CALB1); ATPase plasma membrane Ca^2+^ transporting 1 (ATP2B1); ATPase plasma membrane Ca^2+^ transporting 4 (ATP2B4); Claudin 2 (CLDN2); Tight Junction Protein 1 (TJP1); Tight Junction Protein 2 (TJP2); Tight Junction Protein 3 (TJP3); Solute carrier family 20 member 1 (SLC20A1); Solute carrier family 34 member 2 (SLC34A2).

The expression of the Ca^2+^/H^+^ exchange pump *ATP2B2* was higher in hens aged 23 weeks at 9–10 h PO than at 0–1 h in hens of both ages, and at 18–19 h PO in old hens (*p* < 0.01, Table 2). The expression of the Ca^2+^ specific *CLDN12* was higher in young hens at 9–10 h PO than in all other conditions (*p* < 0.001, Table 2). The expression of *OCLN* was higher at 0–1 h PO in young hens than in old hens at the same stage, and also higher than at 9–10 h PO for both ages (*p* < 0.01, Table 2). This indicated that the variations observed between stages at 23 weeks of age disappeared at 90 weeks of age.

The results for the genes influenced by the PO stage or the age independently are presented in Table 3. The gene expression of the *VDR*, the cation channel *TRPM7*, the Ca^2+^ channel *TRPV2*, the Ca^2+^ specific claudin *CLDN2*, the anchoring protein *TJP3* and the Sodium-phosphate symporters *SLC34A2* decreased with age (*p* < 0.001, *p* < 0.05, *p* < 0.001, *p* < 0.001, *p* < 0.05 and *p* < 0.001, respectively). By contrast, the Ca^2+^ channel *TRPC1*, the Ca^2+^/H^+^ exchange pump *ATP2B4* and the anchoring protein *TJP1* increased with age (*p* < 0.01, *p* < 0.001 and *p* < 0.001, respectively). Only *TRPC1* was affected by the PO stage, its mRNA expression increasing at 0–1 h compared to 18–19 h PO (*p* < 0.01).

### Candidate genes involved in vitamin D activation and regulation

**Figure 6:**
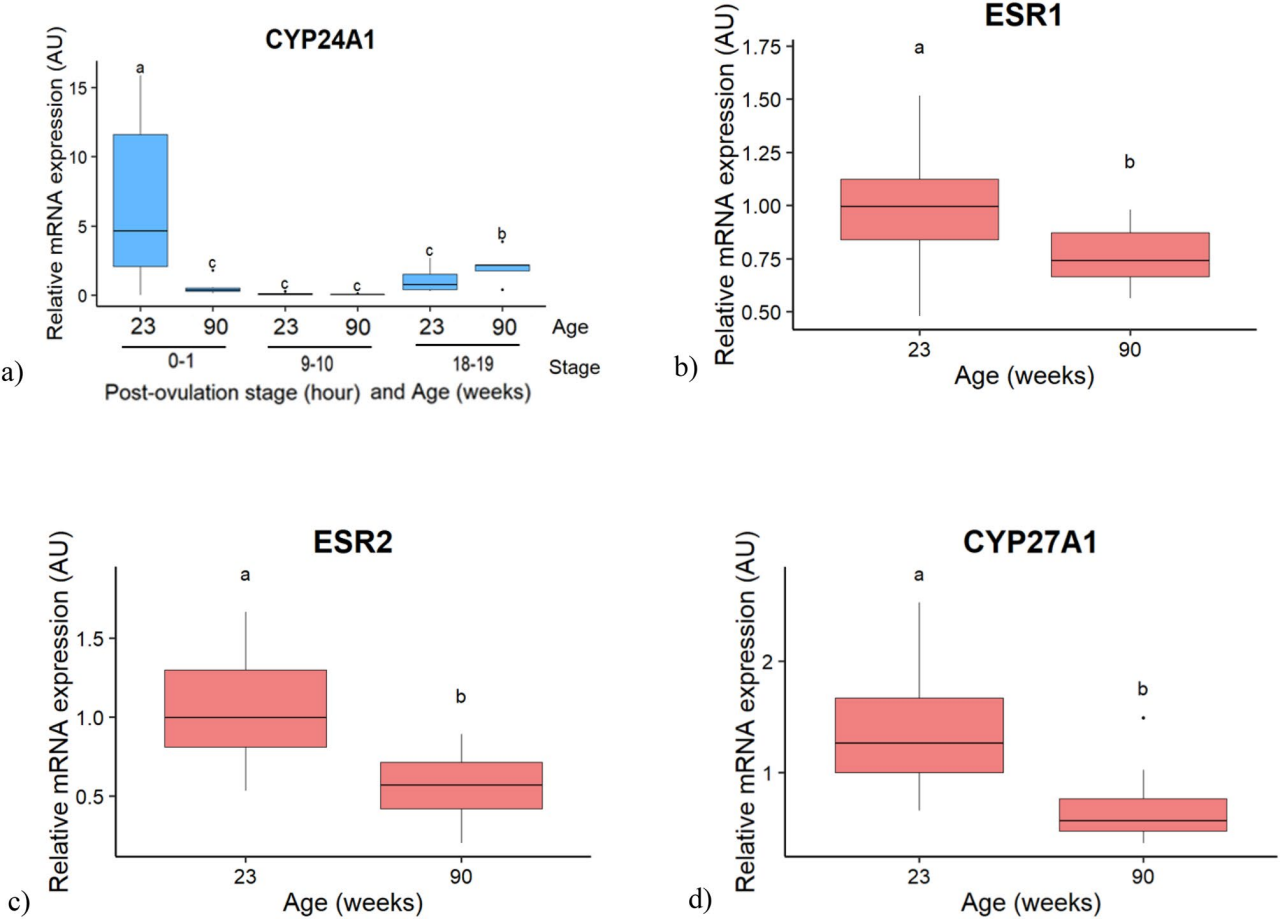
Boxplot of the interaction effect between the post-ovulation stage and the age of hens, or of the age effect on the relative mRNA expression of candidate genes in the kidney (**a**) and in the liver (**b**–**d**). (**a**) Cytochrome P450 family 24 subfamily A member 1 (CYP24A1). (**b**) estrogen receptor 1 (ESR1). (**c**) Estrogen receptor 2 (ESR2). (**d**) cytochrome P450 family 27 subfamily A member 1 (CYP27A1). Values are expressed as Arbitrary Units (AU) with n = 4–7 per group for (**a**) and n = 11–12 per age for (**b**), (**c**) and (**d**). Different letters indicate significant differences (*p* < 0.05).

The expression of *CYP24A1*, encoding an enzyme involved in 24-hydroxylation of vitamin D in the kidney, was affected by the interaction between the PO stage and the age (Fig. 6a; *p* < 0.05). In young hens, the mRNA expression of *CYP24A1* was the highest at 0–1 h PO, then declined at 9–10 h and remained low until 18–19 h PO. In old hens, it was the highest at 18–19 h PO and low at 0–1 h and 9–10 h PO. As a result, the expression level was higher at 0–1 PO and lower at 18–19 h PO in young compared to old hens. The other three candidate genes expressed in the liver, the two estrogen receptors (*ESR1* and *ESR2*) and the 25-hydroxylase *CYP27A1* varied with the age only, decreasing from 23 to 90 weeks of age (Fig. 6b–d ; *p* < 0.05).

## Discussion

Several studies conducted on ageing laying hens reported alterations of Ca metabolism and its regulations, which contributed to osteoporosis and reduced eggshell quality^3,7,12,16,17,20^. In younger hens at maximum of lay, the ovulatory cycle alters the circulating levels of 25(OH)D_3_, 1.25(OH)_2_D_3_ and *i*P, and the expression of several genes involved in Ca and P regulation across tissues^18^. We hypothesized that ageing could alter those regulations.

**Figure 7:**
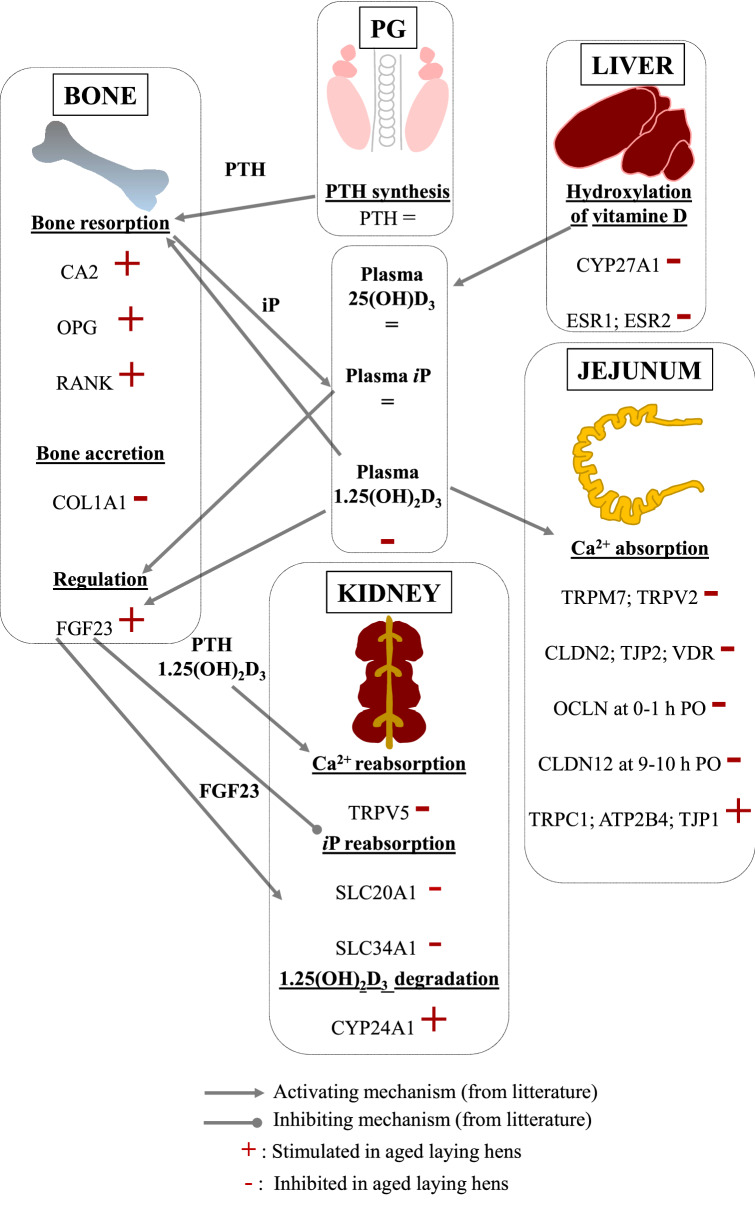
Integrative model describing the effect of ageing on plasma parameters and on the expression of candidate genes regulating calcium metabolism in the parathyroid gland (PG), medullary bone, liver, jejunum and kidney.

The observed effects of age are summarized in the Fig. 7. Of the measured plasma parameters, only 1.25(OH)_2_D_3_ levels markedly decreased with age, in the absence of variation in 25(OH)_2_D_3_ levels, suggesting that either the synthesis of 1.25(OH)_2_D_3_ or its degradation, respectively mediated by the renal 1 hydroxylase and 24 hydroxylase enzymes, could be altered. In the present study, the increase of the 24 hydroxylase (*CYP24A1*) mRNA expression in the kidney occurred earlier in old than in young hens (18–19 h vs 0–1 h), when 1.25(OH)_2_D_3_ was required to stimulate Ca retention and bone resorption^21^. We hypothesize that it could contribute to the lower plasma levels of 1.25(OH)_2_D_3_ observed in old hens_,_ as observed in ageing rats^22^. This is in contrasts with previous studies comparing young and old hens at a single stage of the ovulatory cycle, where no difference of renal 24-hydroxylase activity was reported^16,20^. In the liver, a reduced expression of the 25-hydroxylase enzyme (*CYP27A1*) was observed with ageing, and coincided with that of both oestrogen receptors *ESR1* and *ESR2*. Although no variation of oestrogen plasma levels has been reported with hens ageing^23^, a reduced expression of *ESR1* mRNA and protein has been observed in the uterine gland^24^. A study conducted on rats showed that the activity of the 25-hydroxylase enzyme was linked to the oestrogenic status^25^. Altogether, this suggests a possible causal link between decreased hepatic ESRs and *CYP27A1* expression in the hen. Although 25(OH)D_3_ did not differ with age, this difference in gene expression could contribute to establish a lower vitamin D status in the long term.

In aged laying hens, several genes involved in the transcellular (*TRPM7*, *TRPV2*) and the paracellular Ca^2+^ transport pathways, including *CLDN2* and the anchoring protein *TJP3* in jejunum, were downregulated together with *VDR*. The increase of *ATP2B2* and of *CLDN12* observed at 9–10 h PO in the jejunum of young hens was blunted in older hens, so that *CLDN12* was lower in old hens at this stage. This is consistent with the reported decrease of intestinal Ca absorption in old hens^12,17^. As far as the two situations can be compared, it is worth mentioning that a decrease of intestinal Ca absorption has been reported in ageing human^26^. A decrease of intestinal VDR content and plasma level of 1.25(OH)_2_D_3_ have also been observed with ageing in rats^27^. As the binding of 1.25(OH)_2_D_3_ to VDR controls the expression of genes involved in Ca absorption^28,29^, reduced levels of both may explain the reduced expression of its target genes. Moreover, the Ca transporter *TRPV5*, which drives Ca reabsorption by the kidney^30^ decreased in old hens, which would lead to urinary loss of Ca and further exacerbate the metabolic challenge to its homeostasis.

It is well established that the medullary bone contributes to sustain the Ca^2+^ demand by the uterus^5,6^. The overexpression of *VDR* in the medullary bone of aged hens could contribute to the enhanced bone resorption, given its documented role in osteoclasts formation^31,32^. The OPG/RANKL/RANK system is the major regulator of osteoclastogenesis^33^. In the present study, *RANK* expression increased earlier in old than in young laying hens, while *OPG* expression was higher in old hens, which could limit the access of RANKL to its receptor RANK, and thus its activation. The final consequences of these perturbations on osteoclastogenesis remain to be further studied. Nevertheless, the CA2 enzyme, which triggers acidification at the resorption plate^34^, was overexpressed in old hens, suggesting that the osteoclasts were active to resorb the bone matrix. The intense bone resorption was apparently insufficient to cover the Ca demand by the uterus, resulting in decreased eggshell quality with age in the present, as in previous studies^14,15^. Bone accretion is a crucial step over the ovulatory cycle in hens, necessary for the restauration of Ca and P storage in the bone^5,6,35^. The expression of the most abundant collagenic protein in bone^36^, collagenic protein Type 1 alpha 1 (*COL1A1*) was downregulated in old hens, as observed in postmenopausal osteoporotic women^37^. Altogether, these results highlighted an imbalance in the medullary bone turnover in old laying hens, which could contribute to the reported loss of medullary bone mass^16^.

Bone resorption liberates P at levels which exceed the need for the eggshell synthesis and must therefore be eliminated by the kidney^4,38^. In mice, the urinary elimination of P excess is triggered by the the phosphaturic factor FGF23, which reduces the reabsorption of *i*P by the kidney, and the expression of P transporters mRNA^39^. The present data suggest that the decreased expression of two P renal transporters (*SLC20A1* and *SLC34A1*) in old hens could result from the high levels of FGF23. The decreased expression of the P transporter *SLC34A2* in the jejunum could also result from a similar mechanism, as FGF23 also indirectly decreases P intestinal absorption^40^. Both mechanisms could contribute to maintain phosphatemia within normal ranges.

The marked increase of *FGF23* expression appears as a signature of a deteriorated Ca/P balance in ageing laying hens. We believe that it could be a key driver of the phenotypic differences between these physiological stages. Indeed, one study reported that osteoporosis in postmenopausal women was accompanied by an increased circulating level of FGF23^41^. This could reflect a status of chronic imbalance between Ca demand by the uterine gland and Ca retention. It could also participate to the decrease of 1.25(OH)_2_D_3_ levels in old hens, as FGF23 is known to increase the degradation of 1.25(OH)_2_D_3_ in the kidney^42^. The low plasma levels of the active form of vitamin D_3_ could in turn further reduce Ca retention and increase bone loss in old hens. This is in contrast with the adaptation of young hens to an unfavourable Ca source, which occurs by increasing 1.25(OH)_2_D_3_ levels, in the absence of FGF23 variations^18^. In this case, several genes implicated in bone degradation were also upregulated, as well as genes involved in bone accretion^18^. This suggests that bone turnover was elevated but still equilibrated to maintain eggshell quality with a challenging diet, which obviously was not the case in old hens although fed a favourable diet. Further studies would be required to unravel the primary causes of the increased expression of FGF23 in old hens. This could result from a chronic state of P excess, originating from bone loss or from the diet. Alternatively, as PTH stimulates FGF23 production^43^, the daily stimulation of PTH secretion induced by the demand of Ca by the uterine gland could contribute to establish a state of chronic FGF23 overexpression.

## Conclusion

Overall, this study provides new information regarding the possible origin of osteoporosis in the laying hen, through an imbalance in Ca homeostasis. This results from a decrease of Ca retention, leading to a cumulative loss of bone mass, as the laying hen prioritizes Ca for eggshell formation. This imbalance is exacerbated by the vitamin D deficiency and could be amplified by the chronic over expression of *FGF23*. Further research is needed to test strategies to improve the management of old hens. A first strategy could selectively target circulating FGF23, for example by immunization as previously tested in younger hens^19^. A second strategy could rely upon a better adaptation of dietary P supply to the needs of old hens, saving this nonrenewable resource and limiting the stimulation of FGF23 production.

## Methods

### Ethics statements

The experiment was conducted under the guidelines of the French Ministry of Agriculture for Animal Research at INRAE Poultry Experimental Facility UE PEAT (2018, https://doi.org/10.15454/1.5572326250887292E12). The experimental design was approved by the Regional Ethics Committee on animal experimentation (Tours, France) and the French Ministry of Higher Education and Research (Paris, France; authorization: 10043).

### Experimental design and samples collection

Two groups of 20-week-old and 87-week-old laying hens (Isa Brown strain, Hendrix Genetics Layers, Ploufragan, France) were allocated to individual cages, in order to record the oviposition time for a period of 3 weeks, as described before^17^. They were fed a standard diet supplemented with a mix of 30% fine (below 0.5 mm) and 70% coarse (between 2 and 4 mm) particles of CaCO_3_. The diets were formulated based on nutritional values adapted to the physiological stages. For hens from 20 to 23 weeks of age: 2710 kcal/kg of metabolized energy, 17.3% of proteins 3.5% of total Ca–0.50% of total P and 2000 UI/kg of vitamin D_3_. For hens from 87 to 90 weeks old: 2680 kcal/kg of metabolized energy, 15.4% of proteins 3.5% of total Ca–0.45% of total P and 2000 UI/kg of vitamin D_3_. All animals had free access to water and feed, and were subjected to a photoperiodic program of 14 h light:10 h dark.

Six or seven laying hens per diet and post-ovulation stage (0–1 h, 9–10 h and 18–19 h PO) were submitted to blood collection and subsequently euthanized as described before^18^. Tissue samples (jejunum, medullary bone of the left tibia, left kidney, parathyroid gland and a piece of liver) were collected and stored at − 80 °C until further analyses. The eggs were collected in utero for determination of the precise stage in the ovulatory cycle based on the weight of the dried eggshell as previously described by Nys and coworkers^44^.

### Blood analysis of plasma *i*P and vitamin D metabolites

Total plasma Ca was measured by ICP as before^44^. Plasma *i*P was analyzed using a commercial kit (Kit Phosphore UV 61571; bioMérieux, 69280 Marcy l'Etoile, France)^18^. In addition, determination of 25(OH)D_3_ and 1.25(OH)_2_D_3_ concentrations were performed by a chemiluminescent immunoassay (Diasorin, Saluggia, Italy) at the Liaison XL platform “Service des Explorations Fonctionnelles” (G.H. Necker Enfants Malades, 75743, Paris Cedex 15, France), as previously described^45^.

### Selection of candidate genes and primer design

The candidate and housekeeping (HKGs) genes were those selected and validated for quantification using RTqPCR by Gloux et al.^18^. The list of genes with the corresponding primers designed with the aid of Primer-BLAST^46^ is available as a supplementary Table. The validation of all primers was performed by RT-qPCR as previously described^18,45^.

### RNA extraction and gene expression assay using RT-qPCR

Prior to RNA extraction, samples of medullary bone, kidney, liver and jejunum were ground in liquid nitrogen. All samples were extracted as before^18^ using methods adapted from Chomczynski and Sacchi^47^. For the liver, kidney and jejunum, total RNA was extracted using the RNANow kit (Ozyme, Saint-Quentin en Yvelines, France). The total RNA of medullary bone and parathyroid gland was extracted using the Trizol reagents (TRI Reagent T9424 and TRI Reagent LS T3934, and TRI Reagent T9424 respectively, Sigma Aldrich, St Louis, USA). Concentration and quality of the extracted RNA were assessed and gene expression was conducted using 96.96 Dynamic Array™ IFCs (BMK-M-96.96, Fluidigm®) as previously described^45^.

### Calculation of relative gene expression

The relative mRNA quantification was performed according to ΔΔCt calculation method proposed by Pfaffl^48^. For a given tissue, the relative expression ratio (R) of a candidate gene was calculated, based on the Efficiency (E) and the cycle threshold (Ct) deviation of a cDNA sample (bone, parathyroid gland, kidney, liver, and jejunum of individuals) versus a control (cDNA mix of all samples for each tissue at a similar concentration) and expressed in comparison to the geometric average of a set of HKGs according to the equation:

$${\text{R}} = \left( {1 + {\text{E}}\,{\text{candidate}}} \right)\,\Delta \,{\text{Ct}}\,{\text{candidate}}\,\left( {{\text{control}} - {\text{sample}}} \right)/{\text{Geometric}}\,{\text{average}}\,\left[ {\left( {1 + {\text{E}}\,{\text{HKGs}}} \right)\,\Delta \,{\text{Ct}}\,{\text{HKGs}}\,\left( {{\text{control}} - {\text{sample}}} \right)} \right]$$

The most stable HKGs (out of 9) were chosen for each tissue on this particular set of samples using GeNorm^49^:

- Medullary bone: GAPDH, MATR3, PPIA, STAG2, B2M and EIF3I
- Parathyroid gland: GAPDH, EIF3F, PPIA, EIF3I, MATR3, STAG2 and TBP
- Liver: EIF3I, MATR3, SDHA and TBP
- Kidney: EIF3I, MATR3, SDHA, TBP and STAG2
- Jejunum: GAPDH, EIF3I, SDHA, TBP, MATR3, PPIA and EIF3F

### Egg parameter evaluation

Egg weights were measured immediately after collection (one egg per hen for 10 days). The eggshell breaking strength (N) was measured with an Instron testing machine (Instron model 1102, Hight Wycombe, Bucks, HP12 3SY UK) as the maximum force (N) required to fracture each egg. The fracture toughness (N/m^3^) describes the eggshell’s ability to resist to the expansion of an existing fracture, as described by Bain^50^. The eggshells were subsequently rinsed with water to remove albumen and placed in a desiccator for 24 h to estimate dry shell weight and calculate eggshell percentage = ( eggshell weight/egg weight) × 100.

### Statistical analyses

All statistical analyses were performed using the R 3.4.0 software (R Core Team, 2017, Vienna, Austria). For plasma levels of *i*P, vitamin D metabolites and the mRNA expression of genes in the different tissues, the normality of the data was checked by a quantile–quantile plot, which identified outliers based on the linear relationship between theoretical and sample percentiles. Thereafter, the data were analyzed using a linear model by a robust regression (R Package MASS version 7.3–51.1), which reduced the weight of outliers in the analysis. The effects of the main factors PO stage, age and the interaction were tested. In case of significant effects in the model (*p* < 0.05), all pairwise comparisons were performed using the least-square means (LSMeans) method (R Package emmeans version 1.3.0) and a Tukey adjustment.

To evaluate the eggshell quality parameters, the statistical model was adapted. The normality of the data was checked by a quantile–quantile plot. The corresponding data were analyzed with a linear mixed model (R Package nlme version 3.1-137) including the main factors age and day of egg collection, and their interaction was tested. The hen, considered as the experimental unit, was included as a random effect in the model. In the case of significant effects in the model (*p* < 0.05), all pairwise comparisons were performed using the least-square means (LSMeans) method (R Package emmeans version 1.3.0) and a Tukey adjustment.

## Supplementary information

Supplementary Table.
