# Supplementary material for: Increased expression of fibroblast growth factor 23 is the signature of a deteriorated Ca/P balance in ageing laying hens
Source: Sci Rep. 2020 Dec 3;10:21124. doi: 10.1038/s41598-020-78106-7 (PMC7713066; doi:10.1038/s41598-020-78106-7)
Supplement: Supplementary file 1 — Supplementary Table. [file 41598_2020_78106_MOESM1_ESM.pdf]

Increased expression of Fibroblast Growth Factor 23 is the signature of a deteriorated Ca/P balance in ageing laying hens. A Gloux , N Le Roy, N Même, ML Piketty, D Prié, G Benzoni, J Gautron, Y Nys, A Narcy, MJ Duclos

Gene symbol	Gene Name	Tissue	Function	Forward/ reverse primer (5'--> 3')	Amplicon size (bp)	GeneBank Accession #	Gene ID
CASR	calcium sensing receptor	parathyroid gland	calcium sensing receptor	CCCGAATCAGTGGAGTGCAT/ GCCACAAAACTCAGAGTGGC	185	XM_416491.5	418266
PTH	parathyroid hormone	parathyroid gland	parathyroid hormone	ATCTGGCCAAGGCCATAGTG/ TCTCCACAGTGTGTCGATGC	135	NM_205452.2	396436
VDR	Vitamin D3 receptor	medullary bone/ intestine	Vitamin D_3 receptor	CGATGTTCACCTGTCCGTTC/ CGATGACTTTCTGCTGCTCC	231	NM_205098.1	395988
COL1A1	collagen type I alpha 1 chain	medullary bone	bone accretion	ACCCTAAGACAAAGAGCCCC/ GTTCGGGTTTCCACACATCC	196	XM_015273228.1	395532
ITGB1	integrin subunit beta 1	medullary bone	bone remodeling	GCGTGGTTGCTGGAATTGTT/ ACAGTTGTCACTGCACTCTTG	166	NM_001039254.2	374058
MMP2	matrix metallopeptidase 2	medullary bone	bone remodeling	TGATGCAGCCTTCAACTGGG/ GGTGTACCCGCTGTCAGTAAG	184	NM_204420.2	386583
ACP5	acid phosphatase 5, tartrate resistant	medullary bone	bone resorption	GTTTTTGGCCGTGGGTGATT/ CTCCCAAATCAACCTCCGGG	184	XM_015302697.2	107057619
CA2	carbonic anhydrase 2	medullary bone	bone resorption	ATCGTCAACAACGGGCACTCCTTC/ TGCACCAACCTGTAGACTCCATCC	101	NM_205317.1	396257
CTSK	cathepsin K	medullary bone	bone resorption	TACTACGACACGAGCTGCAA/ TTGTTCATATTGCGGGCCAG	158	NM_204971.2	395818
RANK	receptor activator of NF-κB	medullary bone	enhance osteoclatogenesis	CAGCTCTGGGATTGGCAGAA/ TATTTGGCTGCACACCTCGT	236	XM_004939688.2	769909
RANKL	receptor activator of NF-κB ligang	medullary bone	enhance osteoclatogenesis	GAAGGATGAAGCAGGCCCTC/ GTTTGCCTGGCCTTTGTCAT	236	NM_001083361.1	428067
OPG	osteoprotegerin	medullary bone	inhibit osteoclatogenesis	GCTAGCTCTGCCTGATTTCC/ TGCACATCACCTGACGAGAT	175	NM_001033641.1	378803
FGF23	fibroblast growth factor 23	medullary bone	phosphaturic hormone	CCACCACCATACTCCCAGTT/ CTGGGCACATGGGAAAAGTC	197	XM_425663.3	428104
CYP27A1	cytochrome P450 family 27 subfamily A member 1	liver	25-hydroxylase	CCCAGCACTTCATCGATTCC/ TCCTTGCCCTTCTCTACGTG	111	XM_422056.5	431683
GC	GC, vitamin D binding protein	liver	protein binding vitmain D metabolites	TAGCAACTCACGCCGAACAC/ CATGGCTCGGAAGTCATCCTT	95	NM_204882.1	395696
ESR1	estrogen receptor 1	liver	nuclear receptor for estrogens	GCTGGAATGTGCCTGGTTAG/CATCATCCGAAACCGAGCAG	181	NM_205183.2	396099
ESR2	estrogen receptor 2	liver	nuclear receptor for estrogens	GCCTGTCACCAGAGAGACATTA/CGTGCATTGATTGGTAGCTGG	229	NM_204794.2	395575
CYP27C1*	cytochrome P450 family 27 subfamily C member 1	kidney	1α-hydroxylase	TCGTGGCAGGAATACAGAGA/ ACTGCCACATCTTTGGGTTT	125	XM_422077.2	424227
CYP24A1	cytochrome P450 family 24 subfamily A member 1	kidney	24-hydroxylase	AAACCCTGGAAAGCCTATCG/ CCAGTTTCACCACCTCCTTG	133	AF019142.1	395827
TRPV5	transient receptor potential cation channel subfamily V member 5	kidney	calcium channel	TGGAACGGACTAAGTCAGAAGTTG/ CGTTATGGCTGGGATGTTGTT	141	XM_004938143.2	418307
KL	klotho	kidney	facilitate FGF23 binding with FGFR	ATGGCGATGTCCCGGTTTAT/ AGTGTCGGGACCAGGAAAAC	287	XM_417105.5	418909
FGFR2	fibroblast growth factor receptor 2	kidney	receptor of FGF23	TGCGCAGACAGGTAACAGTG/ GCGTCAGCTTATCCCTTGGA	177	NM_205319.2	396259
FGFR3	fibroblast growth factor receptor 3	kidney	receptor of FGF23	TGCTGAAGACGGCAGGTGTTA/ AAGAGGACTAAGCCAGTGCC	232	NM_205509	396515
SLC20A2	solute carrier family 20 member 2	kidney	Sodium-phosphate symporter	TCTAAAGAAGGAAGACCCCGTG/ CTAAGGACACGCCGAGTGAT	167	NM_001305398.1	422493
SLC34A1	solute carrier family 34 member 1	kidney	Sodium-phosphate symporter	TGGCTAGCTCCTTCCAGATC/ GAGATGCCGAAGATGAGGGA	202	XM_015293844.1	395402
ATP2B1	ATPase plasma membrane Ca^{2+} transporting 1	intestine/kidney	Ca^{2+} /H^{+} exchange pump	CTGCACTGAAGAAAGCAGATGTTG/ GCTGTCATATACGTTTCGTCCCC	146	NM_001168002.3	374244
CALB1	Calbindin 28K	intestine/kidney	intracellular Ca^{2+} binding protein	CAGGGTGTCAAAATGTGTGC/ GCCAGTTCTGCTCGGTAAAG	215	NM_205513.1	396519
SLC20A1	Solute carrier family 20 member 1	intestine/kidney	Sodium-phosphate symporter	AGGGCAGAAAGGCGTCAA/ CGAGGAAGAAGAGAACAGCAGA	104	XM_015297502.2	426250
OCLN	Occludin	intestine	barrier protein of tight junction	CCGTAACCCCGAGTTGGAT/ ATTGAGGCGGTCGTTGATG	214	NM_205128.1	396026
ATP2B2	ATPase plasma membrane Ca^{2+} transporting 2	intestine	Ca^{2+} /H^{+} exchange pump	TTACTGTACTTGTGGTTGCTGTCCC/ GGTTGTTAGCGTCCCTGTTTTG	176	XM_025154762.1	415958
TRPC1	Transient receptor potential cation channel subfamily C member 1	intestine	Ca^{2+} channel	CATCGAGTGGCAAAGTGAAA/ AGTTCGAAAGCCAAGGAGGT	233	NM_001004409.2	424776
TRPV2	Transient receptor potential cation channel subfamily V member 2	intestine	Ca^{2+} channel	ACTTCCCCTCTCTTTGGCTG/ AGTCTTCACACCTGCCTTCA	211	XM_004946685.3	417603
ATP2B4	ATPase plasma membrane Ca^{2+} transporting 4	intestine	Ca^{2+}/H^{+} exchange pump/ BPM	TGCTCTGAAGAAAGCTGATGTTGG/ GCTGGTGAAGTTGTCATCCGTC	103	XM_015298964.2	419934
TRPM7	Transient receptor potential cation channel subfamily M member 7	intestine	cation channel	GTGTTCCCAGGAAGGCAATA/ GCTTGAAGAAATGGGGTCAA	196	NM_001177555.1	427502
CLDN12	Claudin 12	intestine	increase permeability to Ca^{2+}	ACGAGAGGAATGTGACCGTT/ TTGGCACGCTTGATACGAAG	225	XM_025148431.1	771872
CLDN2	Claudin 2	intestine	increase permeability to Ca^{2+}	CGCTCGTATCTCTTGCTTGG/ AGAGTATGGCTGTGACGAGG	185	NM_001277622.1	422292
SLC34A2	Solute carrier family 34 member 2	intestine	Sodium-phosphate symporter	GTCCGTTCACTCTGTTGCCT/ TGGGTCCTCTTCTTGCCTTG	242	NM_204474.2	395131
TJP1	Tight junction protein 1	intestine	tight junction protein connecting transmembrane proteins	ACCGAGAGATGCTGGTACTG/ GCACAGCCTCATTCTCATGG	208	XM_015278981.2	415388
TJP2	Tight junction protein 2	intestine	tight junction protein connecting transmembrane proteins	CATTGTTCGGGAGGATGCTG/ AGCCAGCCAGTTTCCTAGTT	247	NM_204918.1	395751
TJP3	Tight junction protein 3	intestine	tight junction protein connecting transmembrane proteins	GGATACAGTGCGGCAGATTG/ TGGTAGCAGTGAAGAGGTGG	245	XM_015299758.2	420070
B2M	beta-2-microglobulin	all tissues	Housekeeping gene	GATCCCGAGTTCTGAGCTGT/ GCTTGCTCTTTGCCGTCATAC	128	XM_015279077.1	414830
EIF3F	eukaryotic translation initiation factor 3 subunit F	all tissues	Housekeeping gene	CTAACTGCTTCTCCGTCCCG/ ATGTCGTGCCCTGTTGCATA	142	XM_421624.4	423748
EIF3I	eukaryotic translation initiation factor 3 subunit I	all tissues	Housekeeping gene	GACATGTGCTCACTGGCTCT/ CACTGCTGAGCTGGTCTTCA	95	NM_001164395.1	419653
GAPDH	glyceraldehyde-3-phosphate dehydrogenase	all tissues	Housekeeping gene	TCTCTGTTGTTGACCTGACCTG/ ATGGCTGTCACCATTGAAGTC	155	NM_204305.1	374193
MATR3	matrin 3	all tissues	Housekeeping gene	ATTCACAAGGTCATGGGCGT/ CCTTCCAAGAGATGCTGGCA	92	NM_204147.1	373948
PPIA	peptidylprolyl isomerase A	all tissues	Housekeeping gene	CGCTGACAAGGTGCCCATAA/ GTCACCACCCTGACACATGA	124	NM_001166326.1	776282
SDHA	succinate dehydrogenase complex flavoprotein subunit A	all tissues	Housekeeping gene	AGATACGGGAAGGAAGGGGT/ ACCGTAGGCAAAACGGGAAT	169	NM_001277398.1	395758
STAG2	stromal antigen 2	all tissues	Housekeeping gene	GCACACACCAGTCATGATGC/ TGGTGTTCAGGCTGCATAGG	117	XM_004940828	422360
TBP	TATA-box binding protein	all tissues	Housekeeping gene	GCGTTTTGCTGCTGTTATTATGAG/ TCCTTGCTGCCAGTCTGGAC	122	NM_205103.1	395995

he paralogue of CYP27B1 in mammals
